# High-Resolution Ultrasonography of the Superficial Peroneal Motor and Sural Sensory Nerves May Be a Non-invasive Approach to the Diagnosis of Vasculitic Neuropathy

**DOI:** 10.3389/fneur.2016.00048

**Published:** 2016-03-30

**Authors:** Nurcan Üçeyler, Kristina A. Schäfer, Daniel Mackenrodt, Claudia Sommer, Wolfgang Müllges

**Affiliations:** ^1^Department of Neurology, University of Würzburg, Würzburg, Germany

**Keywords:** nerve ultrasonography, vasculitis, sural nerve, superficial peroneal nerve, peripheral neuropathy

## Abstract

High-resolution ultrasonography (HRUS) is an emerging new tool in the investigation of peripheral nerves. We set out to assess the utility of HRUS performed at lower extremity nerves in peripheral neuropathies. Nerves of 26 patients with polyneuropathies of different etiologies and 26 controls were investigated using HRUS. Patients underwent clinical, laboratory, electrophysiological assessment, and a diagnostic sural nerve biopsy as part of the routine work-up. HRUS was performed at the sural, tibial, and the common, superficial, and deep peroneal nerves. The superficial peroneal nerve longitudinal diameter (LD) distinguished best between the groups: patients with immune-mediated neuropathies (*n* = 13, including six with histology-proven vasculitic neuropathy) had larger LD compared to patients with non-immune-mediated neuropathies (*p* < 0.05) and to controls (*p* < 0.001). Among all subgroups, patients with vasculitic neuropathy showed the largest superficial peroneal nerve LD (*p* < 0.001) and had a larger sural nerve cross-sectional area when compared with disease controls (*p* < 0.001). Enlargement of the superficial peroneal and sural nerves as detected by HRUS may be a useful additional finding in the differential diagnosis of vasculitic and other immune-mediated neuropathies.

## Introduction

Diagnosing potentially treatable immune-mediated neuropathies can be challenging, particularly, in vasculitic neuropathy, which is one of the most severe, but treatable forms (1). Vasculitic neuropathy can either be part of a systemic vasculitis or can present as non-systemic vasculitic neuropathy (NSVN) with single organ involvement of the peripheral nervous system (2, 3). Nerve biopsy is the gold standard to prove vasculitic neuropathy (4); however, its sensitivity reaches only 20–60% (1, 4–7). Furthermore, nerve biopsy is invasive, there may be contraindications such as conditions leading to impaired wound healing and potential long-term side effects such as dysesthesias and pain in the biopsy area. Therefore, a non-invasive technique for peripheral nerve assessment is warranted.

High-resolution ultrasonography (HRUS) is a non-invasive technique that is increasingly used in the diagnostic work-up of peripheral neuropathies. Examples of frequently investigated disorders are entrapment syndromes (8), hereditary neuropathies (9), or diabetic neuropathy (10). Recently, also immune-mediated neuropathies have been studied with HURS. Arm nerves are easier to access by HURS (11) but are often less affected in immune-mediated neuropathies, such that sonography of lower limb nerves is increasingly performed (12–14). We prospectively studied the utility of HRUS by assessing lower extremity nerves in patients with immune-mediated and non-immune-mediated neuropathies of different etiologies.

## Patients and Methods

### Case Ascertainment and Study Cohort

Our study was approved by the Würzburg Medical Faculty Ethics Committee (#165/10). All patients gave written informed consent before study inclusion. From November 2010 to July 2012, we prospectively recruited Caucasian patients with neuropathies of different etiologies who came to our Department of Neurology, University of Würzburg, for diagnostic work-up. Patients underwent thorough neurological examination, extensive laboratory tests including cerebrospinal fluid analysis, and electrophysiology was performed adapting to their individual symptoms and signs. Only patients who also underwent sural nerve biopsy for diagnostic reasons were included in our study. Further inclusion criteria were ≥18 years, body height of 160–180 cm. Age- and gender-matched Caucasian disease controls consisted of patients with neurological disorders other than peripheral neuropathies seen at our department for diagnostic and/or therapeutic reasons (e.g., myasthenia gravis, Parkinson’s disease, and transitory ischemic attack). In these patients, peripheral neuropathy was excluded by history and clinical examination; thus, patients with ataxia, distal symmetric hypoesthesia, or peripheral pareses were excluded. Ankle reflexes and vibration sense of at least seven out of eight at the toes were prerequisites for study inclusion of controls. Age was matched with the patient group at a difference of 5 years at a maximum. The following diagnostic subgroups were distinguished.

#### Chronic Inflammatory Demyelinating Neuropathy

Patients were diagnosed as chronic inflammatory demyelinating neuropathy (CIDP) when the INCAT criteria were fulfilled (inflammatory neuropathy cause and treatment) (15).

#### CIDPclin

These patients had the typical clinical presentation and laboratory findings and showed a demyelinating neuropathy in neurophysiological and histological assessment, which is the characteristic for CIDP, but did not fulfill the neurophysiological INCAT criteria.

#### CIDPsens

These patients had purely sensory symptoms with a duration of ≥2 months, signs of demyelination in neurophysiological assessment, signs of demyelination and inflammation in the sural nerve biopsy, elevated cerebrospinal fluid protein, a positive response on steroid treatment, and normal to slightly reduced intraepidermal nerve fiber density in the skin punch biopsy taken from the distal lateral thigh during sural nerve biopsy (see below) (16, 17).

#### Vasculitic Neuropathy

These patients were divided in those with systemic vasculitis and those with NSVN (4).

#### Chronic Idiopathic Axonal Polyneuropathy

These patients reported slow disease onset with slow progression. Clinical presentation was sensory-motor and neurophysiology revealed axonal neuropathy. Histology was axonal but without signs of inflammation. Cerebrospinal fluid was normal and intraepidermal nerve fiber density was reduced. Steroid treatment was inefficacious (18).

#### Unknown Etiology

In this group, we summarized all cases in which a definitive diagnosis as detailed above was not possible at the time point of study inclusion.

In some cases, neuropathy turned out to be associated with amyotrophic lateral sclerosis (ALS), later, which was diagnosed according to the revised El-Escorial criteria (19). One patient had adrenomyeloneuroropathy.

## Diagnostic Analyses

### Laboratory Tests

The following laboratory tests were performed in all patients: whole blood and differential cell counts, chemistry panel, erythrocyte sedimentation rate, C-reactive protein, antinuclear antibodies (ANA), antibodies against extractable nuclear antigen, ENA, antineutrophil cytoplasmic autoantibody (ANCA), rheumatoid factor, HCV antibodies, HBV surface antigen, *Borrelia* antibodies, serum ACE, and in selected cases with abnormalities on immunofixation or immunoelectrophoresis we searched for cryoglobulins. Other causes of neuropathies had been excluded with regard to the individual history and presentation by appropriate laboratory tests (e.g., HbA1c, oral glucose tolerance test, serum electrolytes, immunofixation for monoclonal gammopathy, vitamin B12 levels, and creatine kinase). All patients had undergone diagnostic lumbar puncture, either at our department or in a hospital prior to admission with most of the data available to us. The cerebrospinal fluid was examined for glucose, cell count, protein levels, and oligoclonal bands.

### Neurophysiological Assessment

Neurophysiological assessment was done in all patients following standard procedures (20), including nerve conduction studies of motor and sensory nerves at the lower and upper limbs in combination with needle electromyography in weak muscles. Of all electrophysiological tests performed during routine patient assessment, we only used the results of conduction studies of the sural nerve that was later biopsied and of the tibial nerve (antidromic recording; surface electrodes) for the present analyses. This was due to the heterogeneity of the data obtained during individual patient assessment. Results were compared with the normal values of our department’s electroneurography unit: lower limit of normal for sural nerve sensory nerve action potential (SNAP) amplitude of 10 μV for patients ≤65 years of age, 5 μV for >65 years of age; sural nerve conduction velocity (NCV) of 40 m/s for all adult ages; tibial nerve compound motor action potential (CMAP) of 10 mV, upper limit of distal motor latency (dmL) of 6.0 ms, and lower limit of NCV of 40 m/s for all adults. Motor nerve conduction block was assumed if tibial nerve CMAP on the proximal stimulus was below 50% of distal amplitude in the absence of significant dispersion (<30%). If skin temperature measured at distal calf level was <32°C, legs were warmed up to ≥34°C by water immersion.

### Sural Nerve Biopsy

Diagnostic sural nerve biopsy was performed under local skin and tissue anesthesia excluding the nerve (21) in those cases that were of sufficient severity and progression and where the etiology of neuropathy was unclear after clinical, laboratory, and neurophysiological assessment. Nerve specimens were processed for routine stains (hematoxylin–eosin for overview and nerve morphology, elastica van Gieson for morphology of the vessel wall, and Congo red for amyloid) on paraffin sections, for semithin sections (azure-methylene blue), and for immunohistochemistry for T cells and macrophages (22).

### High-Resolution Ultrasonography

**Figure 1:**
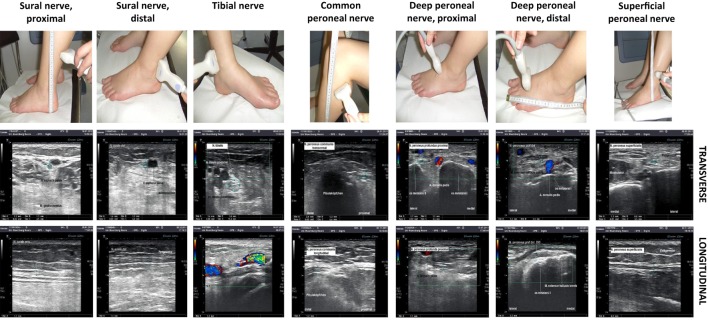
**The photographs in the upper row show the investigation sites of the reported peripheral nerves**. In the middle row, an example for each nerve is given in the transverse sections and in the lower row in longitudinal sections. The nerves are marked with either cyan circles (transverse) or crosses (longitudinal).

For HRUS of the peripheral nerves, the Aplio XG ultrasonography device with an 18 MHz linear transducer was used equipped with its standard Tissue Harmonic Imaging Software (Toshiba Medical Systems, Japan). All assessments were performed unilaterally in a standardized manner on transverse and longitudinal sections in sitting or prone position as appropriate. Device mode was kept the same for all assessments and the zoom function was not used during measurements (23). The investigator (Kristina A. Schäfer) was trained and supervised thoroughly by two experienced ultrasonographers (Wolfgang Müllges and Mira Schließer). All data were additionally cross-checked off-line (Wolfgang Müllges). HURS assessment was performed in a blinded manner as for the exact neuropathy diagnosis. The following nerves were examined and exact landmarks are illustrated in Figure 1:

– sural nerve, 20 cm proximal lateral malleolus and at level of lateral malleolus,
– tibial nerve, at level of medial malleolus,
– common peroneal nerve, 5 cm below head of fibula,
– deep peroneal nerve, 3 cm proximal of lateral malleolus and 15 cm distal of lateral malleolus,
– superficial peroneal nerve, 5 cm above lateral malleolus.

At each measurement point, the following parameters were determined (Figure 1):

– largest transversal diameter (LTD) in millimeter (i.e., diameter upon sonography of the nerve in a transversal section),
– smallest transversal diameter (STD) in millimeter (i.e., diameter upon sonography of the nerve in a transverse section),
– longitudinal diameter (LD) in millimeter (i.e., diameter upon sonography of the nerve in a longitudinal section),
– transversal cross-sectional area (CSA) in square millimeter.

During all measurements, the probe angle was positioned perpendicular to the nerve. All measurements were performed manually within the hyperechoic nerve rim. Two measurements were recorded at each investigated site after reposition of the probe and data were averaged. In patients, nerves were examined on the right side; the sural nerve that was later biopsied was always investigated. In control subjects, the side of examination was selected individually.

### Statistical Analysis

For statistical analyses, IBM SPSS Version 23 was used (Ehningen, Germany). Data with normal distribution in the Shapiro–Wilk test were assessed using the parametric Student’s *t*-test and the Welch test; non-normally distributed data were evaluated with the non-parametric Mann–Whitney *U*-test. *p*-Value <0.05 was assumed as significant. Data are illustrated as box-and-whisker plots displaying the median, the upper 75% and lower 25% percentiles, and the minimum and maximum values.

## Results

### Patient Characteristics, Clinical and Laboratory Presentations

We included 26 consecutive patients with polyneuropathies of different etiologies (20 males and 6 females) and a median age of 66 years (range 37–81 years). The median time between symptom onset and diagnostic evaluation at our department was 2 years (0.06–30 years). The age- and gender-matched group of disease controls consisted of 26 subjects (20 males and 6 females) with a median age of 70 years (range 39–84 years). Table 1 gives demographic data including diagnostic subgroups. For individual neurophysiological data, see Table S1 in Supplementary Material.

**Table 1:** **Patients’ clinical characteristics and diagnostic subgroups**.

Item	Number (% of entire group)
M, F (*N*)	20, 6
Median age (range)	66 years (37–81)
Median disease duration (range in years)	2 years (0.06–30)
Diagnostic subgroups, *N* (% of entire group)	
Unknown etiology	9 (35)
Vasculitic neuropathy	6 (23)
NSVN	4 (67)
ALS	3 (12)
CIDP	2 (8)
CIDPclin	2 (8)
CIDPsens	2 (8)
CIAP	1 (4)
Adrenomyeloneuoropathy	1 (4)

*ALS, amyotrophic lateral sclerosis; CIAP, chronic idiopathic axonal polyneuropathy; CIDP, chronic inflammatory demyelinating polyneuropathy; CIDPclin, patients with a clinical presentation typical of CIDP, but not fulfilling electrophysiological INCAT criteria; CIDPsens, patients with pure sensory clinical presentation and otherwise like CIDP, but not fulfilling all electrophysiological INCAT criteria; F, females; INCAT, inflammatory neuropathy cause and treatment group; M, males; *N*, number; NSVN, non-systemic vasculitic neuropathy*.

### Sural Nerve Histology

Histological assessment of the sural nerve showed evidence of vasculitic neuropathy in 6/26 (23%) patients in accordance with the published criteria (4). Among the remaining cases, non-vasculitic histological signs of inflammation contributed to the diagnoses of CIDP, CIDPclin, CIDPsens, and chronic idiopathic axonal polyneuropathy (CIAP) in 7/26 (27%) patients. These 13 cases were summarized as immune mediated and were compared with the remaining non-immune-mediated cases in further analyses, in addition to the separate analyses of the vasculitis cases.

### Peripheral Neuropathies are Associated with Lower Extremity Nerve Enlargement

**Figure 2:**
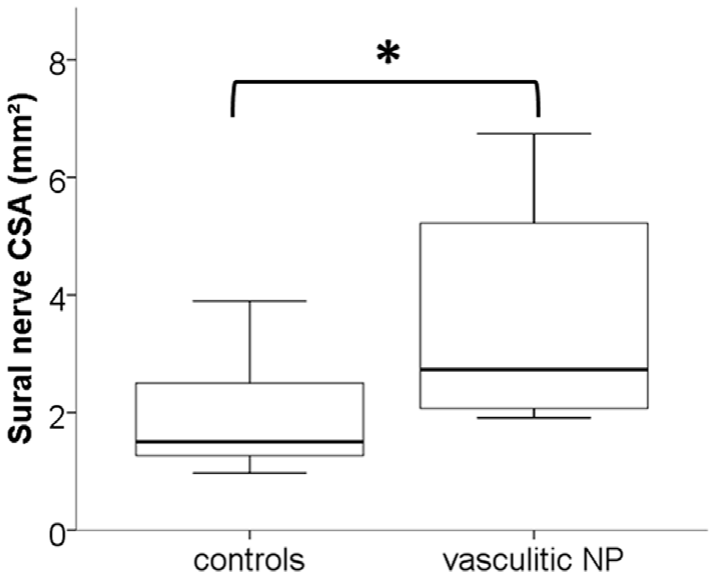
Box-and-whisker plots illustrate the enlarged median cross-sectional area (CSA) in high-resolution ultrasonography (HRUS) of the sural nerves of patients with vasculitic neuropathy compared with disease controls (*p < 0.05).

Table S2 in Supplementary Material summarizes the means and SDs of the obtained HRUS data. Almost all examined nerves and parameters showed higher values for the group of patients with neuropathies compared to controls (Tables S2 and S3 in Supplementary Material). However, most of the determined parameters did not distinguish between immune-mediated and non-immune-mediated neuropathies (Tables S2 and S3 in Supplementary Material). Particularly HRUS data of the later biopsied sural nerve were not different when comparing histologically inflamed- and non-inflamed nerves. This was also true for the group of vasculitic neuropathy, which only showed larger sural nerve CSA when compared with disease controls (*p* < 0.05, Figure 2).

### Superficial Peroneal Nerve HRUS LD is Most Informative When Comparing Diagnostic Subgroups

**Figure 3:**
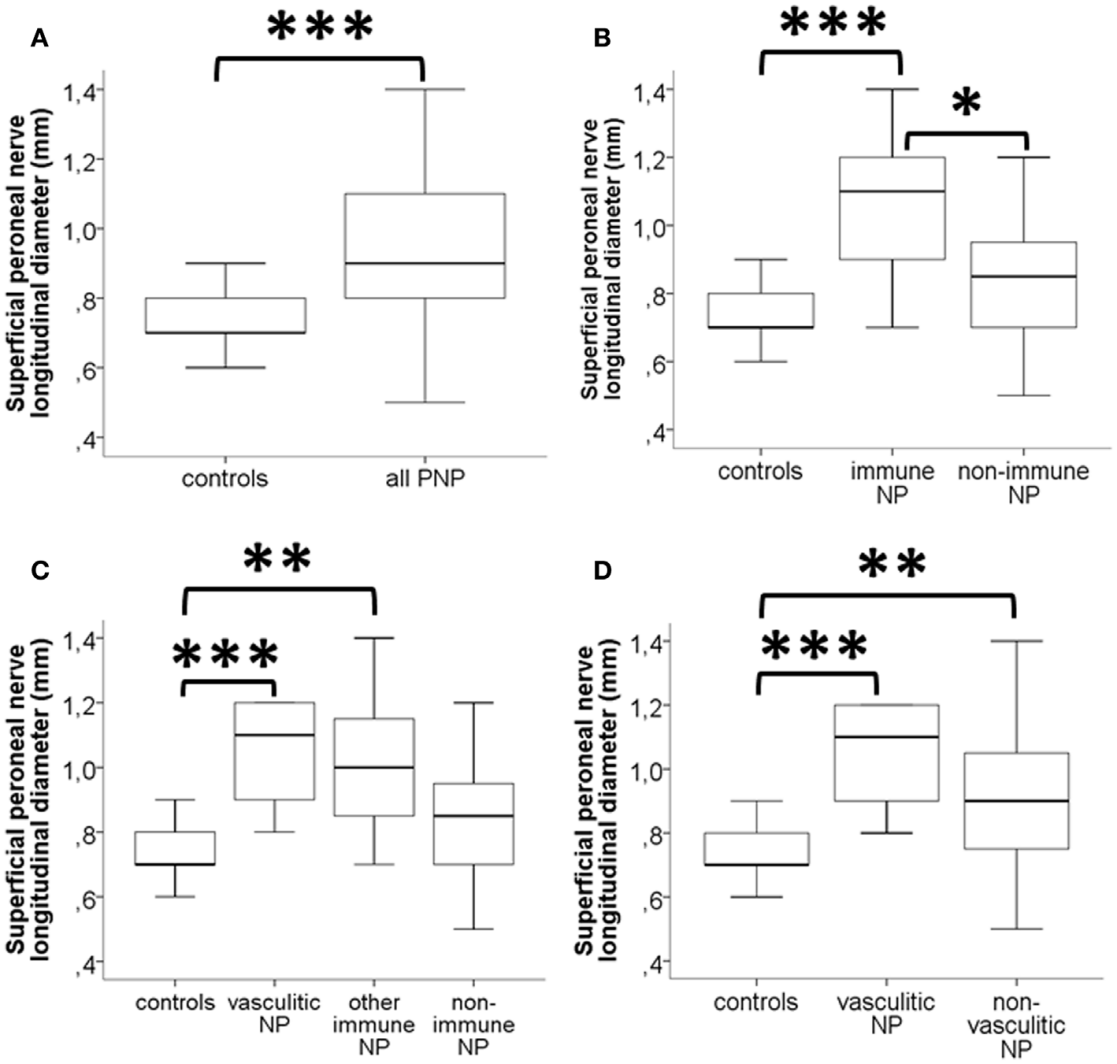
**Box-and-whisker plots illustrate the results of high-resolution ultrasonography (HRUS) of the superficial peroneal nerve in patients with neuropathies and disease controls; the longitudinal diameter (LD) is displayed in millimeter**. **(A)** Patients with peripheral neuropathies have larger LD of the superficial peroneal nerve than disease controls. **(B)** The subgroup of patients with immune-mediated neuropathies (“immune NP”) has increased LD when compared to disease controls and to patients with non-immune-mediated neuropathies. **(C)** When separating vasculitic neuropathy from the group of other immune-mediated neuropathies, LD was largest, and also, when comparing vasculitic neuropathy with all other non-vasculitic neuropathies (“non-immune NP”) **(D)**. *p < 0.05, **p < 0.01, and ***p < 0.001.

The superficial peroneal nerve LD was the most informative parameter when comparing the entire group of neuropathy patients with controls. Median LD was larger in patients with neuropathies compared to disease controls (*p* < 0.001, Figure 3A). The LD of the superficial peroneal nerve did not differ between patients with non-immune-mediated neuropathies and controls but was larger in patients with immune-mediated neuropathies compared to patients with non-immune-mediated neuropathies (*p* < 0.05) and controls (*p* < 0.001, Figure 3B). This was particularly the case in patients with vasculitic neuropathy (Figures 3C,D). See Video S1 in Supplementary Material for a demonstration of the measurement of the superficial peroneal nerve CSA and LD.

## Discussion

We investigated lower extremity nerves of patients with immune-mediated and non-immune-mediated neuropathies of different etiologies and compared data with disease controls and among neuropathy subgroups. We showed that neuropathies lead to an enlargement of lower extremity nerves, in general, and that the superficial peroneal nerve LD may be a useful parameter indicating immune-mediated, and particularly, vasculitic neuropathies.

Our study is one of the few examining nerves of the lower extremities in patients with polyneuropathies of different etiologies. In the majority of studies, upper limb nerves have been assessed due to easier access. However, peripheral neuropathies typically affect lower extremities first and more severely; thus, systematic HRUS investigation of lower limb nerves is essential and increasingly performed (13, 14).

The CSA is regarded as the most precise and reproducible parameter in HRUS (24) and is therefore reported in the majority of studies. When comparing our mean values of nerve CSA with previously published data from other laboratories, we see discrepancies. For instance, mean CSA of the proximal sural nerve was reported as 5.3 mm^2^ (25, 26) or 3.6 mm^2^ (27, 28) in healthy controls by other groups; this is much larger than our mean value of 1.7 mm^2^ and that reported in recent studies (12, 14). For the proximal part of the sural nerve, the mean CSA as reported by Hobson-Webb et al. (5.2 mm^2^) was also larger than in our study (1.9 mm^2^) (26). Similarly, our mean CSA results for the distal tibial nerve were smaller than in previous studies (9, 28–32) but comparable with recent data (12, 14). In patients with vasculitic neuropathy, Ito et al. reported a mean CSA for the sural nerve of 4.9 ± 0.5 mm^2^, which is larger than our data (2.0 ± 1.7 mm^2^) and for the tibial nerve of 13.5 ± 3.7 mm^2^ (30), which is much larger than the 3.5 ± 0.9 mm^2^ found in our study. For the common peroneal nerve, published CSA data are again larger than those determined in our study. Published values range between 11.2 ± 3.3 and 16.1 mm^2^ (26, 31–35), whereas in our study, mean CSA was 4.1 ± 2.3 mm^2^, which is again similar with data in more recent studies (12, 14).

There are several potential reasons for these discrepancies. Major issues are the performance of the ultrasonography devices used and the methodology of measurements. It is of note that, in early studies, less powerful devices with 5–12 MHz transducers were used (33), in contrast to the currently available 15–18 MHz transducers. Moreover, the device itself together with the Tissue Harmonic Imaging algorithm influences the delineation of bordering lines. Also, the manually performed measurements are investigator dependent and a potential source of discrepancy, particularly when using the zoom function (23). Another aspect is the exact site of measurement, which is different between studies. Thus, the comparison of HRUS data across studies is only possible if the measurements are performed under identical conditions. It is also desirable that every sonography laboratory establishes its own normative values (9).

Nerve biopsy is the gold standard for the diagnosis of vasculitic neuropathy (4, 36). The sural nerve, the superficial radial nerve, and the superficial peroneal nerve are potential candidates for diagnostic biopsy in peripheral neuropathies. The reported diagnostic yield of a sural nerve biopsy alone in vasculitic neuropathy varies between 20 and 60% (5). Some studies emphasized a higher diagnostic value of the superficial peroneal nerve (4, 37), particularly in combination with the peroneal brevis muscle (38), whereas other studies reported no superiority of a combined nerve/muscle biopsy (39, 40). So far, no HRUS data had been published for the deep and superficial peroneal nerve in peripheral neuropathies. In our study, the superficial peroneal nerve LD distinguished best between immune-mediated, particularly, vasculitic, neuropathies, and controls.

Our study gives evidence that peripheral nerve LD may be informative when assessing immune-mediated neuropathies. This parameter distinguished best between the subgroups investigated here and was more reliable than the CSA (24, 41). There are also studies showing that the CSA of a peripheral nerve is equally informative as the LD (42–44). However, when investigating the peroneal nerve, the CSA may not be the ideal parameter. Practically, it is almost impossible to achieve a perfect transversal image of the peroneal nerve when it twists around the fibular caput; at such sites the nerve will be visualized more accurately in the longitudinal plane.

In recent publications, scores combining clinical, electrophysiological, and ultrasonography items were used (45, 46). These score approaches may become valuable additions to single nerve ultrasonography parameters; however, they need to be confirmed in studies with large patient cohorts before routine application.

Our study has several limitations. The study cohort was small and particularly the study subgroups consisted of <10 patients each. The control group was not investigated with nerve conduction studies to fully exclude a potential subclinical neuropathy. We did not investigate all nerves bilaterally and due to the potential asymmetric phenotype of peripheral neuropathies, may have missed the most pathological areas of the respective nerves. As in any sonography, the results very much depend on the investigator’s experience. Kristina A. Schäfer was trained and supervised accordingly (by Wolfgang Müllges and Mira Schließer); additionally, all data were confirmed off-line by a second examiner (Wolfgang Müllges). However, data were not reproduced by a third fully independent investigator. Some measurement sites are also areas prone to entrapment syndromes. However, none of the study subjects had clinical signs of nerve entrapment. We also did not control for the body mass index, for which controversial data are available with regard to influence on the HRUS measurements (9, 47). All patients were Caucasian adults of 160–180 cm in height; thus, data correction for ethnicity and height was not necessary (48). It is also to be noted that there was a large range of disease duration in the patient group, which may have an impact on study results.

The major strength of our study is, first, that diagnostic sural nerve biopsy was performed in all patients, confirming vasculitic neuropathy in a subgroup and allowing the comparison of nerve histology and sonography. This has hardly ever been done in prior studies. Second, we investigated lower extremity nerves, which should be much more informative in patients with neuropathies than nerves of the upper limbs.

The lack of standardization of measurements is a major drawback in current nerve sonography applications. For clinical practice, it is also essential that every sonography unit should establish at-site normative values that are obtained under standardized conditions. Here, we used defined points of transducer positioning. Our study shows that it is well possible to systematically depict the morphology of lower leg peripheral nerves by HRUS. Large scale studies with clinically, neurophysiologically, and histologically well characterized patient cohorts are warranted and eventually supplemented by nerve MRI.

## Author Contributions

NÜ: data assessment and manuscript preparation; KS: data collection and data assessment; DM: data collection and data assessment; CS: data assessment and manuscript preparation; WM: study design, data collection, data assessment, and manuscript preparation.

## Conflict of Interest Statement

NÜ has received honoraria for consultancy from Grünenthal GmbH and for presentations from Genzyme Corp., Shire Corp., and Astellas; she has received travel grants from Pfizer Inc., Genzyme Corp., Shire Corp., Astellas, Grünenthal GmbH, and CSL Behring; she has received research support from Grünenthal GmbH. KS and DM report no disclosures. CS has received honoraria for consultancy from Astellas, Baxter, and CSL Behring; she has given educational talks for Baxter, Genzyme, Grifols, Kedrion, and Pfizer. WM has received honoraria for presentations from Boehringer Ingelheim, Bayer Health Care, Toshiba Medical, Medilab Ultrasound, KoMed Education GbR, German Societies of Neurology, NeuroIntensive Care, Neurosurgery, Internal Medicine, Interdisciplinary Intensive Care, Pneumology, and several hospitals.
