# Supplementary material for: High-Resolution Ultrasonography of the Superficial Peroneal Motor and Sural Sensory Nerves May Be a Non-invasive Approach to the Diagnosis of Vasculitic Neuropathy
Source: Front Neurol. 2016 Mar 30;7:48. doi: 10.3389/fneur.2016.00048 (PMC4812111; doi:10.3389/fneur.2016.00048)
Supplement: Supplementary file 1 [file Table_1.DOCX]

Supplementary Table 1: Individual neurophysiological data of study cohort.

|  | **Sural nerve** |  | **Tibial nerve** |  |  |  |
| --- | --- | --- | --- | --- | --- | --- |
|  | **SNAP [µV]** | **NCV (m/s)** | **CMAP proximal [mV]** | **CMAP distal [mV]** | **dmL** | **NCV** |
| **NSVN** | No potential |  | 0.2 | 0.2 | 5.6 | 39.3 |
| **NSVN** | 5.2 | 41.1 | 3.2 | 4.9 | 7.9 | 35.9 |
| **NSVN** | No potential |  | 0.1 | 0.1 | 5.3 | 31.0 |
| **NSVN** | No potential |  | 0.1 | 0.1 | 9.0 | 23.1 |
| **Systemic vasculitis** | 5.4 | 39.0 | 0.15 | 0.2 | 6.4 | 39.2 |
| **Systemic vasculitis** | No potential |  | 0.1 | 0.9 | 4.1 | 32.5 |
| **CIDP** | 7.2 | 45.5 | 0.5 | 0.9 | 4.4 | 39.1 |
| **CIDP** | No potential |  | 14.7 | 18.4 | 4.4 | 43.8 |
| **CIDPsens** | 4.9 | 30.6 | 5.5 | 10.9 | 4.2 | 33.3 |
| **CIDPsens** | 8.0 | 40.1 | 7.8 | 10.7 | 4.9 | 32.8 |
| **CIDPclin** | 3.5 | 43.9 | 1.2 | 5.2 | 3.3 | 37.9 |
| **CIDPclin** | 8.3 | 37.3 | 11.2 | 20.76 | 3.7 | 33.6 |
| **CIAP** | 3.4 | 45.7 | 10.0 | 17.0 | 3.2 | 45.9 |
| **ALS** | 2.2 | 38.7 | 0.2 | 0.3 | 4.5 | 34.8 |
| **ALS** | 2.3 | 34.9 | 0.2 | 0.3 | 5.2 | 31.7 |
| **ALS** | 6.4 | 40.5 | 0.8 | 1.2 | 4.6 | 35.8 |
| **Adrenomyeloneurophathy** | 8.0 | 40.1 | 7.8 | 10.7 | 4.9 | 32.8 |
| **unknown** | 1.6 | 43.9 | No potential |  |  |  |
| **unknown** | 2.8 | 41.7 | 1.1 | 1.1 | 4.4 | 45.3 |
| **unknown** | No potential |  | 0.7 | 0.6 | 5.7 | 34.8 |
| **unknown** | 4.4 | 44.4 | 16.5 | 26.2 | 3.0 | 51.2 |
| **unknown** | No potential |  | 0.4 | 0.4 | 6.0 | 30.5 |
| **unknown** | No potential |  | 0.1 | 0.1 | 5.6 | 34.6 |
| **unknown** | No potential |  | 0.02 | 0.04 | 5.2 | 31.7 |
| **unknown** | 2.3 | 54.1 | 0.4 | 0.5 | 4.7 | 40.2 |
| **unknown** | 5.9 | 45.5 | 7.8 | 10.4 | 4.5 | 40.2 |
