# Supplementary material for: High-Resolution Ultrasonography of the Superficial Peroneal Motor and Sural Sensory Nerves May Be a Non-invasive Approach to the Diagnosis of Vasculitic Neuropathy
Source: Front Neurol. 2016 Mar 30;7:48. doi: 10.3389/fneur.2016.00048 (PMC4812111; doi:10.3389/fneur.2016.00048)
Supplement: Supplementary file 2 [file Table_2.DOCX]

**Supplementary Table 2:** Mean and standard deviations of high-resolution ultrasonography measurements.

|  | **Controls (n=26)** |  | **Vasculitic neuropathy (n=6)** |  | **All other neuropathies (n=20)*** |  |
| --- | --- | --- | --- | --- | --- | --- |
|  | **Mean** | **SD** | **Mean** | **SD** | **Mean** | **SD** |
| **Sural nerve proximal** | | | | | | |
| LTD [mm] | 1.44 | 0.61 | 1.87 | 0.55 | 1.77 | 0.55 |
| ST [mm] | 0.92 | 0.23 | 1.38 | 0.39 | 1.12 | 0.36 |
| LD [mm] | 0.92 | 0.19 | 1.35 | 0.34 | 1.16 | 0.32 |
| CSA [mm²] | 1.69 | 0.87 | 2.03 | 1.70 | 3.04 | 4.51 |
| **Sural nerve distal** | | | | | | |
| LTD [mm] | 1.59 | 0.41 | 2.03 | 0.62 | 1.89 | 0.45 |
| ST [mm] | 1.04 | 0.23 | 1.55 | 0.60 | 1.30 | 0.39 |
| LD [mm] | 0.98 | 0.25 | 1.40 | 0.23 | 1.23 | 0.30 |
| CSA [mm²] | 1.91 | 0.87 | 3.27 | 2.20 | 2.60 | 1.35 |
| **Tibial nerve** |  |  |  |  |  |  |
| LTD [mm] | 3.28 | 0.74 | 3.53 | 0.93 | 3.62 | 1.11 |
| ST [mm] | 1.47 | 0.41 | 1.68 | 0.62 | 1.65 | 0.48 |
| LD [mm] | 1.27 | 0.23 | 1.78 | 0.56 | 1.64 | 0.48 |
| CSA [mm²] | 5.51 | 2.28 | 5.23 | 2.34 | 5.71 | 2.85 |
| **Common peroneal nerve** | | | | | | |
| LTD [mm] | 3.85 | 0.74 | 3.65 | 0.82 | 4.01 | 1.10 |
| ST [mm] | 1.22 | 0.33 | 1.47 | 0.39 | 1.42 | 0.31 |
| LD [mm] | 1.17 | 0.21 | 1.60 | 0.49 | 1.50 | 0.38 |
| CSA [mm²] | 4.12 | 2.27 | 4.69 | 1.43 | 7.06 | 8.10 |
| **Deep peroneal nerve distal** | | | | | | |
| LTD [mm] | 1.03 | 0.14 | 1.45 | 0.30 | 1.29 | 0.31 |
| ST [mm] | 0.79 | 0.11 | 1.12 | 0.23 | 1.00 | 0.23 |
| LD [mm] | 0.81 | 0.13 | 1.15 | 0.27 | 1.08 | 0.30 |
| CSA [mm²] | 0.76 | 0.10 | 1.24 | 0.38 | 1.03 | 0.37 |
| **Deep peroneal nerve proximal** | | | | | | |
| LTD [mm] | 1.06 | 0.15 | 1.38 | 0.26 | 1.41 | 0.40 |
| ST [mm] | 0.80 | 0.12 | 1.12 | 0.26 | 1.00 | 0.30 |
| LD [mm] | 0.84 | 0.13 | 1.13 | 0.27 | 1.08 | 0.32 |
| CSA [mm²] | 0.82 | 0.13 | 1.20 | 0.37 | 1.17 | 0.51 |
| **Superficial peroneal nerve** | | | | | | |
| LTD [mm] | 1.81 | 0.78 | 1.95 | 0.38 | 2.15 | 0.87 |
| ST [mm] | 0.73 | 0.16 | 1.13 | 0.22 | 0.96 | 0.24 |
| LD [mm] | 0.75 | 0.13 | 1.03 | 0.19 | 0.94 | 0.22 |
| CSA [mm²] | 1.80 | 1.27 | 1.92 | 0.62 | 2.17 | 1.59 |

**Abbreviations:** LTD: largest transverse diameter; STD: smallest transverse diameter; LD: longitudinal diameter; CSA: cross sectional are.

* All other neuropathies: this group consists of patients with amyotrophic lateral sclerosis (n=3), chronic inflammatory demyelinating polyneuropathy (CIDP; n=2), CIDPclin (i.e. typical clinical presentation of CIDP but not fulfilling the diagnostic INCAT criteria; n=2), CIDPsens (i.e. primarily sensory CIDP; n=2), chronic inflammatory axonal polyneuropathy (CIAP, n=1), and adrenomyeloneuoropathy (n=1).
