# Supplementary material for: High-Resolution Ultrasonography of the Superficial Peroneal Motor and Sural Sensory Nerves May Be a Non-invasive Approach to the Diagnosis of Vasculitic Neuropathy
Source: Front Neurol. 2016 Mar 30;7:48. doi: 10.3389/fneur.2016.00048 (PMC4812111; doi:10.3389/fneur.2016.00048)
Supplement: Supplementary file 3 [file Table_3.DOCX]

**Supplementary Table 3:** P-values of group comparisons for HRUS measurements.

|  | **All PNP (n=26) vs all Co (n=26)** | **Vasc (n=6) vs all Co (n=26)** | **All non vasc (n=20) vs all Co (n=26)** | **All non-immune mediated (n=13) vs all Co (n=26)** | **All non-immune mediated (n=13) vs all immune mediated (n=13)** | **All vasc (n=6) vs all non-immune mediated (n=13)** |
| --- | --- | --- | --- | --- | --- | --- |
| **Sural nerve proximal** | | | | | | |
| LTD [mm] | **0.005** | **0.012** | **0.022** | 0.100 | 0.852 | 0.213 |
| ST [mm] | 0.056 | **0.000** | 0.430 | 0.485 | 0.437 | **0.024** |
| LD [mm] | **0.006** | **0.001** | 0.054 | **0.018** | 0.060 | 0.053 |
| CSA [mm²] | 0.079 | 0.055 | 0.210 | 0.676 | 0.979 | 0.335 |
| **Sural nerve distal** | | | | | | |
| LTD [mm] | **0.015** | **0.029** | 0.051 | 0.411 | 0.137 | 0.291 |
| ST [mm] | **0.005** | **0.007** | **0.029** | 0.114 | 0.123 | 0.151 |
| LD [mm] | **0.002** | **0.001** | **0.022** | **0.035** | 0.087 | 0.102 |
| CSA [mm²] | **0.022** | **0.016** | 0.090 | 0.230 | 0.205 | 0.102 |
| **Tibial nerve** |  |  |  |  |  |  |
| LTD [mm] | 0.341 | 0.308 | 0.506 | 0.087 | 0.437 | 0.964 |
| ST [mm] | 0.210 | 0.494 | 0.236 | 0.093 | 0.936 | 0.820 |
| LD [mm] | **0.002** | **0.007** | **0.009** | **0.023** | 0.852 | 0.151 |
| CSA [mm²] | 0.891 | 0.689 | 0.723 | 0.114 | 0.979 | 0.616 |
| **Common peroneal nerve** | | | | | | |
| LTD [mm] | 0.480 | 0.832 | 0.450 | **0.001** | 0.110 | 0.682 |
| ST [mm] | **0.017** | 0.078 | **0.037** | 0.164 | 0.650 | 0.494 |
| LD [mm] | **0.001** | **0.005** | **0.004** | 0.066 | 0.574 | 0.250 |
| CSA [mm²] | **0.034** | 0.087 | 0.076 | **0.035** | 0.689 | 0.892 |
| **Profound peroneal nerve distal** | | | | | | |
| LTD [mm] | **0.000** | **0.000** | **0.008** | 0.297 | 0.247 | 0.125 |
| ST [mm] | **0.001** | **0.002** | **0.006** | 0.016 | 0.040 | 0.151 |
| LD [mm] | **0.000** | **0.005** | **0.003** | 0.146 | 0.650 | 0.750 |
| CSA [mm²] | **0.002** | **0.029** | **0.008** | 0.376 | 0.347 | 0.494 |
| **Profound peroneal nerve proximal** | | | | | | |
| LTD [mm] | **0.000** | **0.000** | **0.004** | 0.100 | 0.728 | 0.250 |
| ST [mm] | **0.012** | **0.003** | 0.084 | 0.485 | 0.247 | 0.151 |
| LD [mm] | **0.007** | **0.003** | **0.047** | **0.018** | 0.852 | 0.437 |
| CSA [mm²] | **0.001** | **0.000** | **0.017** | 0.676 | 0.110 | **0.041** |
| **Superficial peroneal nerve** | | | | | | |
| LTD [mm] | 0.121 | 0.225 | 0.190 | 0.411 | 0.538 | 0.616 |
| ST [mm] | **0.000** | **0.000** | **0.004** | 0.114 | 0.077 | **0.024** |
| LD [mm] | **0.000** | **0.000** | **0.006** | **0.035** | **0.030** | **0.041** |
| CSA [mm²] | 0.276 | 0.285 | 0.418 | 0.230 | 0.728 | 0.682 |

**Abbreviations:** Co: disease control; CSA: cross sectional area; HURS: high-resolution ultrasonography; LD: longitudinal diameter; LTD: largest transverse diameter; PNP: polyneuropathy; STD: smallest transverse diameter; Vasc: vasculitic neuropathy.
